# Health Tract, No. 118

**Published:** 1865

**Authors:** 


### CHILDREN'S EATING.

This is a subject of literally vital interest to every family in the land; more especially in large towns and cities, where the want of facilities and inducements to out-door activities makes it absolutely indispensable to adopt some system in reference to the times, quantities, and qualities of the food to be taken by children; for the want of attention to which things multitudes die early, while other multitudes, not as large however—for half of all that are born die before the age of eighteen years, in consequence mainly of inattention to the habits and health—become dyspeptic, scrofulous, or consumptive before the age of twenty-five, many of whom are destined to a life of weariness, of painful toil, and of wasting efforts for a living through sickness, and disease, and chronic sufferings.

On entering the fifth year, or sixth at farthest, a child can be very easily habituated to eat at three regular times a day, at intervals of five or six hours, with nothing whatever between, except, at a little past mid-way, a single good ripe apple, or a piece of cold, dry, coarse bread may be allowed to the less vigorous. Frequent eatings, at two or three hours' interval, especially in connection with being in the house most of the time, initiates many children into a life-long dyspepsia, simply because the stomach, being kept at work all the time, has no rest, loses its tone and strength, like an over-worked servant or animal, and wears out prematurely.

A second consideration is quantity. If children are taught to eat slowly, in loving good-nature—as will be the case if they are let alone by their parents, and not put in an ill-humor by incessant reprimands and innumerable rules and regulations about a hundred and one contemptible trifles — they may generally be allowed, for breakfast and dinner, to eat as long and as much as they want, only if all the hard food is cut up carefully with a sharp knife into pieces not larger than a pea. This should be conscientiously and always attended to by one of the parents, for it can not be safely intrusted to one hireling out of a million; parental affection only will do it as it ought to be done.

At supper, children should always be controlled; let observation determine how much a child will eat and leave something over, and then allow thereafter certainly not over two thirds of that amount.

And now as to that most important of all items — quality of food for growing children. The instinct for sweetness is inappeasable; without it, any child, however healthy, will soon die, and, fortunately, the two things which children most love every where, and of which they never would get tired, and will always relish when hungry, are milk and bread, and these furnish as much sugar as any child needs. But no child can ever grow up healthy and handsome without good teeth, and as the permanent ones begin to be made from the fourth year, their food should contain in great abundance those elements which are needed for sound, durable teeth. The bony part of the tooth contains seventy-one per cent of lime, the enamel ninety-four per cent. Out of one hundred parts of the finest, whitest flour, only six per cent is lime; of one hundred parts of flour made of the whole grain, there is twenty-five per cent of lime, or four times as much; and no other general article of food contains any thing like as much lime as common brown bread. Therefore, it is a reasonable conclusion that if children were to live largely on flour made of the whole product of the grain, in the shape of well-made and well-baked brown bread, very much would be done toward securing them durable and beautiful teeth. When children are from home, let them live as others; when at home their bread should be uniformly made of the whole product of the grain ground, from their third to their fifteenth year, to be eaten with half a pint of milk for breakfast and supper, adding some berries from June until September, and one or two baked apples the remainder of the year, adding a teaspoon or two of sugar. Such a supper or breakfast will always "taste good" to them. Such a bill of fare, with two or three variations a week, and allowing them to eat what they want for dinner, will pretty surely, other things being equal, give good health, good teeth, a good constitution, and a good old age.

